# Precise intervention of drug-coated balloons based on quantitative flow ratio: treatment efficacy and virtual planning adaptability evaluation

**DOI:** 10.1186/s12872-026-05681-4

**Published:** 2026-03-03

**Authors:** Qingmin Zhou, Xinyuan Zhao, Hangzhou Luo

**Affiliations:** 1 School of Clinical Medicine, Hangzhou Normal University, Hangzhou, People’s Republic of China; 2 Affiliated Hospital of Hangzhou Normal University, Hangzhou, People’s Republic of China

**Keywords:** Quantitative flow ratio, Virtual DCB, Residual quantitative flow ratio, Percutaneous coronary intervention

## Abstract

**Objective:**

To evaluate the efficacy of drug-coated balloon (DCB) treatment for coronary artery stenosis guided by quantitative flow ratio (QFR) and the clinical applicability of virtual balloon planning.

**Methods:**

A single-center retrospective study was conducted on 48 patients (52 lesions). Changes in minimum lumen diameter (MLD) and QFR values before and after treatment were examined. Paired testing and the Bland–Altman method were employed to assess the consistency between the virtual balloon size (length/diameter) and actual size used in clinical practice.

**Results:**

MLD and QFR values improved significantly after DCB treatment (MLD: 1.20 ± 0.40 mm to 2.15 ± 0.50 mm, *P* < 0.001; QFR: 0.78 ± 0.14 to 0.94 ± 0.04, *P* < 0.001), with a complication rate of 1.92%. The virtual balloon length was significantly shorter than the actual length (15.65 ± 11.88 mm vs. 20 ± 8 mm, *P* < 0.001). No significant bias was observed in the balloon diameter (2.55 ± 0.6 mm vs. 2.75 ± 0.5 mm, *P* = 0.443), although the 95% limits of agreement were wide (–0.70 to 0.78 mm), with a clinical concordance rate (± 0.5 mm) of 84.91%.

**Conclusion:**

QFR-guided DCB treatment effectively improves hemodynamic and anatomical parameters with good short-term safety. However, virtual balloon planning algorithms require optimization to enhance clinical applicability and the accuracy of balloon size prediction.

## Introduction

Currently, percutaneous coronary intervention (PCI) primarily relies on drug-eluting stents (DES) as the main approach, but late-term complications following stent implantation, including in-stent restenosis (ISR), neoatherosclerosis, and stent thrombosis, have garnered significant attention [1–3].To address these clinical challenges, drug-coated balloons (DCBs) have emerged and evolved, offering an interventional therapy modality without implantation [4]. DCBs eliminate the need for permanent implants, reduce the duration of dual antiplatelet therapy (DAPT), and promote late lumen enlargement, vascular remodeling, and regression of atherosclerosis-induced plaques during mid- to long-term follow-up [5]. Although DCBs were recommended primarily for treating ISR in bare-metal stents (BMS) or DES [6], recent studies have demonstrated their safety and efficacy in various conditions, including small and large vessel disease and complex lesions [7, 8].

Fractional flow reserve (FFR) is the gold standard for physiologically assessing the functional status of nonobstructive coronary arteries [9]. However, its clinical uptake remains limited, possibly because operators often rely on angiographic visual assessment. In addition, FFR requires the use of vasodilators and pressure wires, which increases the economic burden on patients and the risk of complications [10]. Based on three-dimensional coronary angiography reconstruction and computational fluid dynamics, quantitative flow ratio (QFR) has been developed as an alternative to FFR measurement [11]. QFR does not employ pressure wires or additional medications [12] and can accurately predict post-PCI pressure outcomes across different stent positions and lengths [13]. Studies conducted in China [14], Japan, and Europe [15] have confirmed the feasibility of online QFR in assessing the structural and functional aspects of coronary stenosis compared with FFR. Furthermore, Murray’s law-based QFR (µQFR) allows the evaluation of coronary stenosis from a single angiographic view, demonstrating excellent feasibility and diagnostic accuracy [10]. The FAVOR III China study further confirmed that, compared to standard angiography-guided strategies, QFR-guided PCI for lesion selection improves 1-year clinical outcomes [16]. In this study, we aim to evaluate the effectiveness and safety of DCB in treating coronary stenosis using QFR while further validating the feasibility of QFR-guided DCB therapy in this setting.

## Materials and methods

### Study design and population

This study is a single-center retrospective study, including patients who underwent elective coronary heart disease procedures in the Department of Cardiology at the Affiliated Hospital of Hangzhou Normal University from January 2022 to December 2023. A total of 48 participants were enrolled. The following inclusion criteria were applied: (1) age 18–80 years; (2) coronary angiography displayed severe coronary stenosis (≥ 70%); (3) meeting the indications for DCB therapy (based on DCB guidelines or expert consensus); (4) DSA imaging is complete, and the pre- and post-operative imaging perspectives are essentially identical. The exclusion criteria included the following: (1) lesions in the left main coronary artery, in-stent restenosis, bifurcation lesions, acute or chronic total occlusions, severely calcified or tortuous arteries, or diffuse lesions with one or more additional lesions deemed angiographically significant or insignificant; (2) history of coronary artery bypass grafting; (3) presence of severe heart failure (NYHA ≥ III), liver or renal insufficiency, malignant tumors, acute or chronic infections, severe coagulopathy, pregnancy, or refusal to sign an informed consent. This study was conducted in accordance with the Declaration of Helsinki and was approved by the Ethics Committee of Hangzhou Second People’s Hospital (Approval No.2022(E2)HS-052). All participants provided their informed and signed consent forms.

### Baseline data collection

Patient information in this study was obtained from the hospital’s electronic medical record system, including gender, age, smoking history, history of hypertension, diabetes, dyslipidemia, and stroke. The lesion vessels were classified by anatomical location, including the left anterior descending artery (LAD), left circumflex artery (LCX), and right coronary artery (RCA). Based on the vessel diameter, the lesions were categorized as small vessels (diameter ≤ 2.75 mm) or large vessels (diameter > 2.75 mm) [17]. Follow-up was conducted through telephonic interviews or with reference to outpatient and imaging records. Telephonic follow-up was performed to assess whether patients experienced recurrent symptoms such as chest pain or underwent any additional procedures. The outpatient and imaging records were used to evaluate the most recent post-procedural data so as to determine the restenosis rate (stenosis ≥ 70%) and the number of patients requiring repeat revascularization.

### QFR assessment

**Fig. 1:**
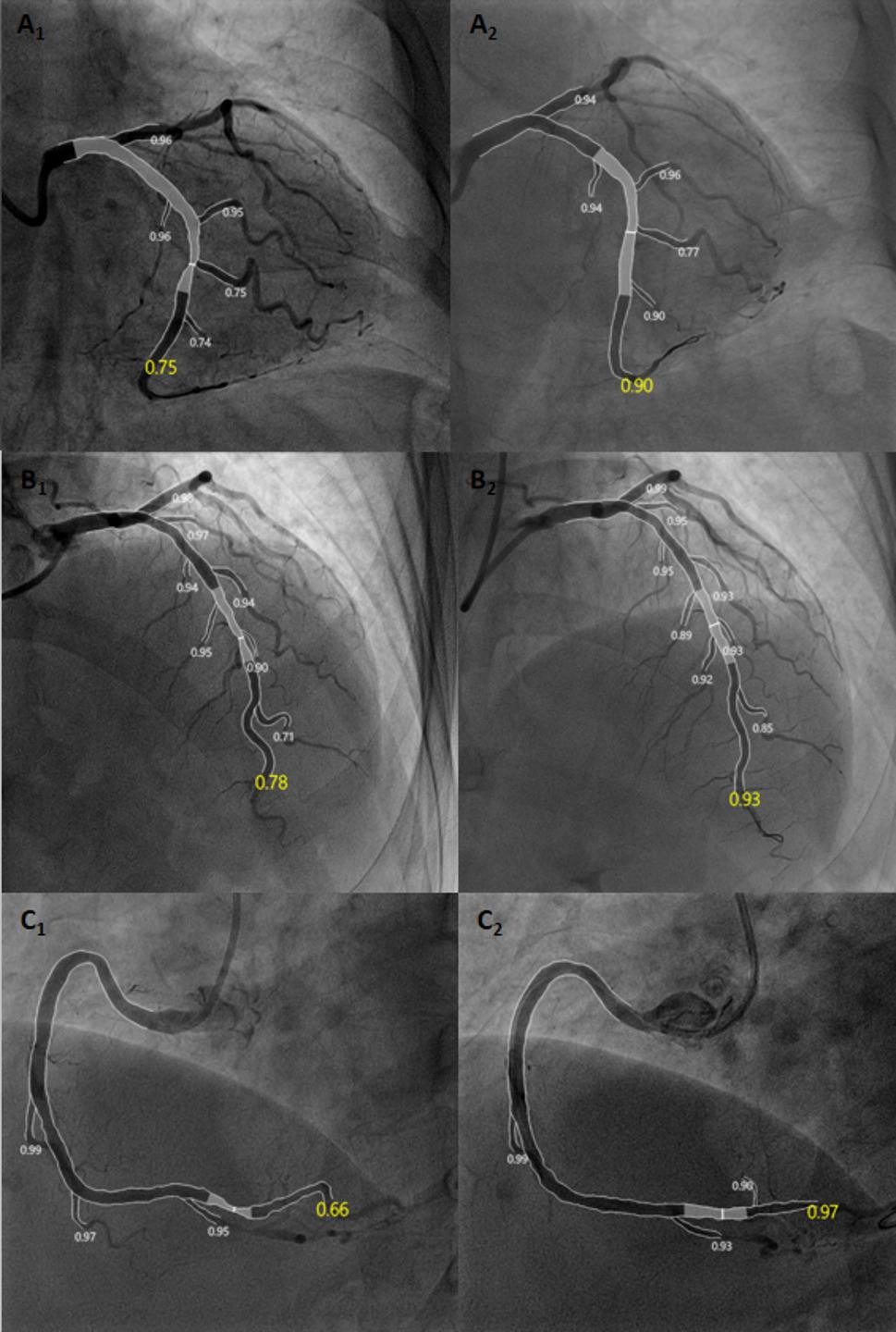
QFR Measurements Before and After Drug-Coated Balloon Angioplasty, Images **A**, **B** and **C** represent lesions in the LCX, LAD and RCA respectively. **A**_1_, **B**_1_, **C**_1_ Pre-procedure: The QFR values are 0.75, 0.78, and 0.66 respectively, indicating a significant hemodynamic impairment at the target lesion, suggesting reduced coronary flow reserve warranting intervention. **A**_2_, **B**_2_, **C**_2_ Post-procedure: The QFR values are 0.90, 0.93, 0.97 respectively, demonstrating significant hemodynamic improvement, approaching the normal range (QFR ≥ 0.80 is typically considered indicative of normal flow reserve), confirming effective restoration of vascular function by drug-coated balloon (DCB) angioplasty

Following enrollment based on coronary angiography outcomes, the QFR system software (AngioPlus Galley, Pulse Medical Imaging Technology Co., Ltd., Shanghai, China) was used to evaluate the following parameters: minimum lumen diameter (MLD), percentage of maximum stenosis area (AS%), target lesion segment length, proximal diameter, distal diameter, pre-procedural QFR value (pre-QFR), residual QFR value (residual-QFR), difference between pre-QFR and residual-QFR, post-procedural QFR value (post-QFR), and changes in post and pre-QFR (ΔQFR). Acute lumen gain was calculated by subtracting pre-procedural MLD from post-procedural MLD. For QFR calculation: Analysts selected a single projection angle of coronary angiography images with the clearest exposure of the target lesion, free from foreshortening or overlap. The system, based on artificial intelligence, automatically identified the vessel type and tracked the target lesion trajectory, precisely delineating the vessel lumen contour (excluding artifacts and interference from branch vessels). AI synchronously detected the contrast agent filling path and calculated its flow velocity as input parameters for the fluid dynamics model. When combined with 3D quantitative coronary angiography (3D-QCA) reconstruction outcomes, the system first calculated an empirical fixed µQFR (fQFR) value using a fixed flow velocity mode, followed by a contrast-corrected µQFR (cQFR) value obtained through the frame-density correction method. Finally, based on Murray’s fractal law, µQFR values for the main vessel and side branches were generated [18](Fig. 1 for a case example). All measurements were performed pre- and post-procedure. The difference between the post-procedural and pre-procedural cQFR values was applied to quantitatively assess the acute improvement in hemodynamics. In addition, the feasibility of QFR-guided DCB therapy was evaluated through parameters in PCI planning.

### DCB treatment

In accordance with the guidance of QFR imaging data, the DCB surgical operation was performed:

#### Balloon pre-dilation

First, it was necessary to use ordinary balloons (semi-compliant), non-compliant balloons, or notched balloons (including spike balloons, cut balloons, or double-wire balloons) for pre-dilation at a balloon/vessel diameter ratio of 0.8–1.0. If no severe, flow-limiting dissection (i.e., type C or lower dissection according to the National Heart, Lung, and Blood Institute of the United States) was observed after pretreatment, the procedure proceeded to DCB dilation.

#### DCB dilation

To prevent geographical deficiencies from fully covering the lesion segment, both ends of the balloon should extend at least 2 mm beyond the lesion segment or the pre-treatment segment of the lesion. The ratio of the diameter of the DCB to the blood vessel was selected to be 0.8–1.0. When performing DCB expansion, a pressure of 8–12 atmospheres was applied and maintained for > 30 s.

#### Immediate post-operative assessment

If at least two vertical positions of angiography immediately after the operation showed residual stenosis of ≤ 30% and the QFR derived from coronary angiography images was ≥ 0.80, the operation was considered successful. Meanwhile, complications such as vascular dissection or elastic retraction were recorded. If the effect was not satisfactory after DCB expansion due to severe residual stenosis or dissection, remedial stent implantation was performed.

### Statistical analysis

Statistical analyses were performed using IBM SPSS Statistics 26 and SPSSAU software, with a two-sided significance level set at α = 0.05. *P* < 0.05 was considered to indicate statistical significance. Continuous variables were tested for normality using the Shapiro–Wilk test. Normally distributed data were expressed as the mean ±standard deviation (x̄ ±s), whereas the non-normally distributed data were presented as the median ±interquartile range [M(Q1, Q3)]. Categorical variables were reported as percentages (*n*%). Differences in hemodynamic parameters (µQFR) and anatomical parameters (MLD) pre- and post-procedure were analyzed using paired samples t-test or Wilcoxon signed-rank test. The correlation and agreement between the virtual planned balloon sizes and actual balloon sizes used were assessed using Pearson’s correlation and Bland–Altman analysis, and the correlation coefficient (*r*), mean difference (bias), and 95% limits of agreement (LoA) were calculated. By setting a clinically acceptable threshold, the compliance rate (i.e., the proportion of lesions with differences within the clinically acceptable threshold) was calculated to further evaluate the feasibility of PCI planning in DCB treatment.

## Results

### Clinical characteristics of study subjects

The median age of the 48 study subjects was 65.5 ± 15 years, with 31 males and 17 females. Fifty-two diseased vessels were analyzed. Four patients had two lesions in different vessels. Based on the anatomical location, the distribution was as follows: 24 left anterior descending (LAD) arteries, 15 left circumflex (LCX) arteries, and 13 right coronary arteries (RCA), with LAD lesions accounting for the highest proportion (46.15%). Based on the vessel diameter, there were 22 large vessels and 30 small vessels, with small vessel lesions being the most prevalent (53.85%). Risk factors associated with the lesions included cerebral infarction (22.92%), hypertension (62.50%), Type 2 diabetes (18.75%), hyperlipidemia (35.42%), and smoking (39.58%). The proportion of male patients with a history of smoking was significantly higher than that of female patients (*P* < 0.001), whereas no significant differences were observed in age or other risk factors (Table 1).

**Table 1:** Clinical characteristics of the study subjects [x̄ ± s, *n*%]

Characteristics	Male	Female	Value	*t*-value	*P*-value
Age	61.55 ± 10.02	66.29 ± 9.66		-1.59	0.119
Cerebral Infarction	5(16.1%)	6(35.3%)	2.283		0.131
Hypertension	17(54.8%)	13(76.5%)	2.192		0.139
Type 2 diabetes	7(22.6%)	2(11.8%)	0.843		0.359
Hyperlipidemia	9(29.0%)	8(47.1%)	1.560		0.212
Smoking history	18(58.1%)	1(5.9%)	12.501		< 0.001

### Validation of efficacy and safety of DCB treatment

Offline QFR analysis was conducted on 52 diseased vessels, revealing highly significant differences in MLD and QFR before and after DCB treatment (*P* < 0.001). Further subgroup analysis based on the anatomical location and vessel diameter showed highly significant differences in QFR before and after treatment (*P* < 0.001; Table 2). Among the 48 patients, only 1 experienced a postprocedural complication (Type B dissection), accounting for 1.92% (< 5%) of cases, which was considered a low-probability event. All patients were followed up through outpatient visits, telephone consultations, and imaging assessments. Imaging follow-up records were available for 23 patients (47.92%), with a median imaging follow-up duration of 344 days. Among these, three patients experienced restenosis, representing 13.04% of patients with imaging follow-up and 6.25% of all followed-up patients.

**Table 2:** Changes in MLD and QFR pre- and post-DCB [x̄ ± s, M(Q1,Q3)]

Parameter	Pre-DCB	Post-DCB	Changes post and Pre-DCB	*P*-Value
*n* = 52 diseased vessels				
MLD	1.20 ± 0.40	2.15 ± 0.50	1.00 ± 0.45	< 0.001
QFR	0.78 ± 0.14	0.94 ± 0.04	0.14 ± 0.15	< 0.001
Classified by Coronary Artery (QFR)				
LAD	0.75 ± 0.12	0.92 ± 0.10	0.17 ± 0.10	< 0.001
LCX	0.78 ± 0.10	0.92 ± 0.02	0.14 ± 0.10	< 0.001
RCA	0.78 ± 0.12	0.96 ± 0.02	0.18 ± 0.12	< 0.001
Classified by the diameter of vessels (QFR)				
Large vessels	0.77 ± 0.11	0.95 ± 0.03	0.16 ± 0.12	< 0.001
Small vessels	0.77 ± 0.10	0.93 ± 0.04	0.15 ± 0.08	< 0.001

*MLD* minimum lumen diameter; small vessels: diameter ≤ 2.75 mm; large vessels: diameter > 2.75 mm

### PCI planning

**Fig. 2:**
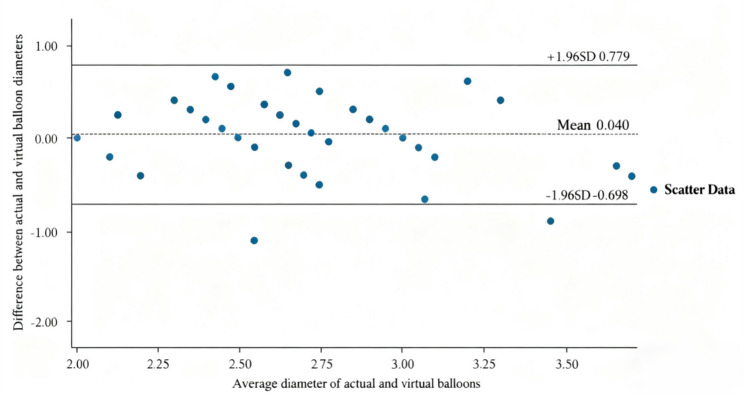
Bland–Altman consistency analysis of virtual planning balloon diameter vs. actual DCB diameter

This study conducted virtual PCI planning based on QFR for 52 lesions and compared the planned parameters (residual-QFR, estimated lesion length, and estimated lesion diameter) with the corresponding parameters after actual post–DCB treatment (post-QFR, DCB length, and DCB diameter). Statistical analysis revealed that, except for balloon diameter, there were highly significant differences between the virtual planning estimates for residual-QFR and lesion length and the actual measured values (*P* < 0.001; Table 3). For balloon diameter, the difference between the virtual planning balloon diameter (corresponding to estimated lesion diameter) and actual DCB diameter was not statistically significant (*P* ≥ 0.05), with a moderate positive correlation between the two (Pearson *r* = 0.61, *P* < 0.001). However, Bland–Altman consistency analysis indicated limited agreement, with a wide 95% limits of agreement range (− 0.698 mm to 0.779 mm) and a standard deviation of 0.38 mm, suggesting non-negligible random error (Fig. 2). Using ± 0.5 mm as the clinically acceptable threshold for diameter difference, the compliance rate (i.e., the proportion of lesions with differences within this range) was 84.91%.

**Table 3:** Corresponding parameters of QFR-based PCI virtual planning and actual DCB treatment outcomes [x̄±s, M(Q1,Q3)]

Parameter	PCI Virtual Planning	Actual DCB Treatment	Difference	*P*-value
QFR	0.97 ± 0.03	0.94 ± 0.04	0.03 ± 0.04	< 0.001
Balloon Length	15.65 ± 11.88	20 ± 8	5.25 ± 7.43	< 0.001
Balloon Diameter	2.55 ± 0.6	2.75 ± 0.5	0.04 ± 0.38	0.443

## Discussion

This study evaluated the effectiveness of QFR-guided DCB treatment for coronary artery stenosis, showing highly significant statistical differences before and after treatment, regardless of the anatomical location or vessel diameter. Multiple observational studies, randomized controlled trials (RCTs), and meta-analyses have confirmed the safety and efficacy of QFR-guided DCB treatment for coronary artery stenosis [19–21].

### Efficacy validation

The immediate postoperative MLD improvement observed in this study was 1.00 ± 0.45 mm, similar to the results reported by Li et al. (1.01 ± 0.10 mm) [19] and Lin et al. (0.94 ± 0.34 mm) [22], indicating a clear lumen expansion effect following DCB treatment. However, the QFR improvement in this study (0.14 ± 0.15) was lower than that reported by Li et al. (0.25). This discrepancy can be reasonably explained by the distinct patient selection criteria in our retrospective study. In our center, the treatment strategy (DCB compared with DES) was influenced by lesion severity and operator judgment. Consequently, patients with more complex or severe lesions tended to receive DES, leading to a DCB cohort with a relatively higher baseline QFR (pre-QFR: 0.78 ± 0.14 compared with 0.71 ± 0.13 in Li et al.‘s study). A higher starting point inherently limits the absolute magnitude of QFR improvement achievable. Furthermore, our post-procedural QFR (0.94 ± 0.04) reached a level comparable to other studies, indicating that a satisfactory hemodynamic result was successfully achieved in this selected population. Therefore, the smaller ΔQFR likely reflects stricter patient selection and a conservative DCB use strategy at our institution rather than inferior treatment efficacy.

### Safety assessment

Previous research has shown a restenosis rate of 11.6% at 9 months in the DCB group [19]. In this study, the restenosis rate at a median follow-up of 11 months was 13.04%, consistent with the expected mid-term safety of DCB treatment. This result affirms the mid-term safety of DCB treatment. It should be noted, however, that the 13.04% restenosis rate was derived from the cohort of patients who underwent follow-up imaging assessment. This rate is higher than the 6.25% observed in the overall follow-up population, a difference that may be attributed to the retrospective study design and the fact that the imaging cohort does not fully represent all enrolled patients. This lower overall incidence of complications may also reflect the conservative patient selection strategy at our center, whereby DCB was preferentially used in lower-risk cases, as discussed in the preceding section. Imaging follow-up records were comprehensive, and only three patients experienced restenosis during this period. One of these patients underwent stent implantation, and the other two continued with DCB treatment. Compared with studies by Lin et al. (with an average angiographic follow-up period of 8.57 ± 1.61 months), the proportion of target lesion revascularisation reached 4.6%, whereas in our study this proportion attained 6.25%. This may be attributable to our longer follow-up duration; for instance, among the three patients undergoing target vessel revascularisation, two had undergone surgery more than 11 months prior. Additionally, one patient developed a type B aortic dissection postoperatively, although no imaging records were available, highlighting the need for more precise individualized treatment plans in clinical practice.

### Case analysis

In this study, three cases of restenosis were observed, with follow-up intervals of 71, 358, and 477 days. Residual-QFR, virtual balloon length, and diameter were evaluated in PCI planning, and the findings showed that residual-QFR and virtual balloon length cannot be directly applied in clinical practice. This discrepancy can be attributed to several factors:

1. The system-generated lesion locations during QFR PCI planning were not adjusted. Instead, the system-generated lesion lengths (at the same surgical site) and the actual DCB lengths were compared.
2. In clinical practice, physicians determine appropriate balloon lengths based on individual patient characteristics, including the presence of bifurcations, calcifications, lipid plaques, and unstable plaques.
3. Different balloon brands with varying length specifications may also contribute to variations between virtual and actual balloon lengths. Shorter balloons in virtual planning produced higher QFR values, likely because QFR assessment in virtual PCI assumes an idealized state of complete stent implantation without residual stenosis, whereas actual post-DCB reflects procedural outcomes (with residual stenosis ≤ 30% considered successful).

Current multicenter RCTs suggest that QFR-guided virtual PCI techniques outperform conventional PCI, but these studies have primarily focused on DES [23, 24]. Research on DCB treatment remains limited, warranting further prospective evaluation. Although there was no statistically significant difference in balloon diameter between virtual planning and actual use (*P* = 0.443), two key issues emerged:

1. The moderate correlation (*r* = 0.61) suggests that virtual planning provides predictive trends rather than precise values.
2. Bland–Altman analysis revealed wide limits of agreement (−0.70 to 0.78 mm), indicating significant random errors. Notably, in extreme deviation cases (−1.1 mm, −0.9 mm), one patient experienced restenosis 71 days postoperatively, highlighting the clinical risks of prediction errors.

In summary, QFR-guided DCB treatment yielded favorable results, achieving immediate lumen expansion and demonstrating acceptable mid-term safety. Although virtual PCI planning offers promising predictive insights, current discrepancies and random errors limit its direct clinical applicability. Future large-scale, multicenter studies with longer follow-up, incorporation of multimodal imaging, and algorithm optimization are needed to refine QFR-guided strategies and establish their role in precise coronary intervention. 

### Limitations

This study has several limitations that should be acknowledged. Firstly, its single-center, retrospective design and small sample size (52 vessels) may limit statistical power and generalizability; however, these findings will inform a future randomized controlled trial. Secondly, lesion assessment relied solely on QFR, precluding intravascular imaging such as intravascular ultrasound (IVUS) to maintain a non-invasive focus; future studies should combine these modalities for a definitive evaluation.Thirdly, this study explored the application of QFR-based virtual PCI planning for DCB therapy, an area less established than for DES. While the virtual planning demonstrated referential value for procedural strategy—showing superior performance in estimating balloon diameter over length—its overall accuracy remains suboptimal. This limitation primarily stems from an idealized simulation environment that overlooks key procedural factors. Therefore, while instructive, a strategy fully reliant on QFR-guidance for DCB is not yet mature. Finally, the median follow-up duration of 11 months may be insufficient to fully capture long-term outcomes; further studies with extended follow-up are warranted to validate the durability of the findings.

## Conclusion

This study demonstrates that QFR-guided DCB angioplasty yields satisfactory post-procedural outcomes. The integrated virtual planning serves as a valuable adjunct, with its diameter assessment showing promise for clinical application. Nevertheless, its length estimation must be complemented by physician judgment and is not a standalone solution. Enhancing the precision of these virtual measurements will therefore remain a central focus for future iterations of the technology.

## Data Availability

The datasets generated and analyzed during the current study are available within the article. Further inquiries can be directed to the corresponding author upon reasonable request.

## Abbreviations

DCB: Drug-coated balloon
DES: Drug-eluting stents
MLD: Minimum lumen diameter
PCI: Percutaneous coronary intervention
ISR: In-stent restenosis
DAPT: Dual antiplatelet therapy
BMS: Bare-metal stents
FFR: Fractional flow reserve
QFR: Quantitative flow ratio
µQFR: Murray’s law-based QFR
fQFR: Empirical fixed µQFR
cQFR: Contrast-corrected µQFR
LAD: Left anterior descending artery
LCX: Left circumflex artery
RCA: Right coronary artery
AS%: Percentage of maximum stenosis area
3D-QCA: 3D quantitative coronary angiography
